# Comparison of the treatment practice and hospitalization cost of percutaneous coronary intervention between a teaching hospital and a general hospital in Malaysia: A cross sectional study

**DOI:** 10.1371/journal.pone.0184410

**Published:** 2017-09-05

**Authors:** Kun Yun Lee, Wan Azman Wan Ahmad, Ee Vien Low, Siow Yen Liau, Lawrence Anchah, Syuhada Hamzah, Houng-Bang Liew, Rosli B. Mohd Ali, Omar Ismail, Tiong Kiam Ong, Mas Ayu Said, Maznah Dahlui

**Affiliations:** 1 Department of Social and Preventive Medicine, Faculty of Medicine, University of Malaya, Kuala Lumpur, Malaysia; 2 Division of Cardiology, Department of Medicine, Faculty of Medicine, University of Malaya, Kuala Lumpur, Malaysia; 3 Pharmaceutical Services Division, Ministry of Health, Selangor, Malaysia; 4 Department of Pharmacy, Queen Elizabeth 2 Hospital, Sabah, Malaysia; 5 Clinical Research Centre, Queen Elizabeth 2 Hospital, Sabah, Malaysia; 6 Department of Pharmacy, Sarawak General Hospital Heart Centre, Sarawak, Malaysia; 7 Administrative Office, Penang General Hospital, Penang, Malaysia; 8 Division of Cardiology, Queen Elizabeth 2 Hospital, Sabah, Malaysia; 9 Department of Cardiology, National Heart Institute, Kuala Lumpur, Malaysia; 10 Division of Cardiology, Penang General Hospital, Penang, Malaysia; 11 Department of Cardiology, Sarawak General Hospital Heart Centre, Sarawak, Malaysia; Kurume University School of Medicine, JAPAN

## Abstract

**Introduction:**

The increasing disease burden of coronary artery disease (CAD) calls for sustainable cardiac service. Teaching hospitals and general hospitals in Malaysia are main providers of percutaneous coronary intervention (PCI), a common treatment for CAD. Few studies have analyzed the contemporary data on local cardiac facilities. Service expansion and budget allocation require cost evidence from various providers. We aim to compare the patient characteristics, procedural outcomes, and cost profile between a teaching hospital (TH) and a general hospital (GH).

**Methods:**

This cross-sectional study was conducted from the healthcare providers’ perspective from January 1^st^ to June 30^th^ 2014. TH is a university teaching hospital in the capital city, while GH is a state-level general hospital. Both are government-funded cardiac referral centers. Clinical data was extracted from a national cardiac registry. Cost data was collected using mixed method of top-down and bottom-up approaches. Total hospitalization cost per PCI patient was summed up from the costs of ward admission and cardiac catheterization laboratory utilization. Clinical characteristics were compared with chi-square and independent t-test, while hospitalization length and cost were analyzed using Mann-Whitney test.

**Results:**

The mean hospitalization cost was RM 12,117 (USD 3,366) at GH and RM 16,289 (USD 4,525) at TH. The higher cost at TH can be attributed to worse patients’ comorbidities and cardiac status. In contrast, GH recorded a lower mean length of stay as more patients had same-day discharge, resulting in 29% reduction in mean cost of admission compared to TH. For both hospitals, PCI consumables accounted for the biggest proportion of total cost.

**Conclusions:**

The high PCI consumables cost highlighted the importance of cost-effective purchasing mechanism. Findings on the heterogeneity of the patients, treatment practice and hospitalization cost between TH and GH are vital for formulation of cost-saving strategies to ensure sustainable and equitable cardiac service in Malaysia.

## Introduction

Epidemiological transition has seen drastic industrialization and lifestyle changes in low- and middle-income countries (LMIC) over the past few decades, leading to an increasing prevalence of cardiovascular disease (CVD). As a rapidly developing middle-income country, Malaysia is not spared of the same epidemic. In 2013, CVD was one of the top 5 causes of hospitalizations and accounted to 24.7% of total mortality in Malaysia [1]. Among the various types of CVD, for example coronary artery diseases (CAD), cerebrovascular disease, peripheral artery disease, congenital heart disease and heart failure, CAD is the most prevalent and it accounts for the highest mortality.

Over the years, developments in modern technology and pharmaceutical devices have led to tremendous revolution in CAD management. The mainstay of treatment included coronary artery bypass graft (CABG), percutaneous coronary intervention (PCI), and fibrinolysis. CABG is often reserved for severe CAD due to its invasive nature. Between the two non-surgical treatment modalities, PCI showed higher success of revascularization and lower complication rates of non-fatal myocardial infarction, stroke and mortality when compared to pharmacological reperfusion [2, 3]. However, PCI is expensive when taking account into the capital cost of cardiac catheterization laboratory and human resource costs of highly skilled staff. Furthermore, costs of PCI consumables such as cardiac stents are escalating with the development of newer generation drug eluting stents (DES) and bio-absorbable vascular scaffolds (BVS). In 2011, a report from the United States showed that PCI with DES insertion accounted for over $5 billion in estimated costs, making it one of the top ten contributors to the healthcare costs [4]. In Malaysia, we saw a similar increasing trend in DES use, which now accounted for 64% of all stents as reported in the National Cardiovascular Disease Database [5].

Despite the high cost, many developing countries, including Malaysia have stepped up the effort to establish PCI service in view of the proven clinical effectiveness. It was first introduced in 1983. By 2007, approximately 9000 PCI procedures were being performed annually at local public and private hospitals, majority being elective cases conducted in public cardiac centres [6]. Public centres providing PCI service in Malaysia included tertiary-level general hospitals under the purview of Ministry of Health and university teaching hospitals of Ministry of Higher Education. Cost differences between teaching or non-teaching institutions have been reported. Teaching hospitals often have greater resource intensity and consequently higher cost of patient care due to their large scale teaching and research programs [7, 8]. For example, a comparison study in United States revealed that patients with myocardial infarction admitted to teaching hospitals were more likely to undergo interventional procedures such as PCI, and incurring higher hospital charges compared to those treated at non-teaching hospitals [9]. However, most of the research findings on comparison between these two entities were based on high-income countries and the general consensus is that patient characteristics, severity of diagnosis and subsequent management often play a role in determining the eventual costs of hospitalizations at teaching hospitals [10].

To date, there is a paucity of research on the resource utilization and hospitalization costs among different hospital system in Malaysia, likewise in other LMIC. Many of the relevant evidence were published by developed countries of different disease profile and health system from Malaysia. Elective PCI, a common cardiac procedure that involved only minor variation between patients, serves as a suitable choice to analyze the variation in terms of clinical practice and resource utilization between different cardiac centres. In the backdrop of escalating healthcare expenditure, reliable clinical and cost evidence are essential for efficient budget allocation and service expansion. In this study, we compared the patient profile, treatment practices, resource utilization and hospitalization costs of cardiac service provision between a teaching hospital (TH) and a general hospital (GH) in Malaysia. These findings will provide important guidance for multiple stakeholders including policy makers and healthcare professionals for long-term financial planning.

## Methods

This study is registered in the National Medical Research Register of Malaysia and received ethical approval from Medical Research and Ethics Committee, of the Ministry of Health (ID: NMRR-13-1403-18234 IIR). Ethical approval was also obtained from the Medical Ethics Committee of University of Malaya Medical Centre, Kuala Lumpur (IRB Reference number: 1038.19).

### Study design

We conducted a cross sectional costing study among patients admitted for PCI at two tertiary-level cardiac centres from January 1^st^ to June 30^th^ 2014. Clinical data was retrieved from a national cardiac registry and primary cost data collection was conducted from the perspective of healthcare providers.

### Study centres

Both study centres are government-owned public hospitals financed via annual budget allocation from the Central Treasury. Labour cost of hospital staffs are paid by the central government agency of public service. Separate budget provisions are made for specific consumables such as the cardiac stents and catheters used in PCI, based on procedural volume from previous years.

TH, located in the central region of West Malaysia, is an academic teaching hospital with undergraduate and postgraduate medical faculty. GH is a state-level tertiary referral hospital situated in the most populous city in East Malaysia. It has a daycare centre for patients who are suitable for same-day discharge after interventional cardiology procedure. Both TH and GH provide full-fledged cardiology and cardiothoracic services and serve as the cardiac referral centres for the region they are located in. In addition to PCI, the cardiac catheterization laboratory in both centres conduct various other procedures such as primary and rescue PCI service, pacemaker insertion, rotational atherectomy, laser atherectomy and intravascular ultrasound. TH employs 12 cardiologists, with five being interventional cardiologists; compared to seven cardiologists stationed in GH, with one being interventional cardiologist. In this study, we presumed that the variations caused by physician’s factors were minimal and negligible as there are up-to-date clinical practice guidelines for the selection of treatment modality and stent usage.

### Patient population

For this study, we included only patients who received elective PCI within the same cardiac centre from admission to discharge. Patients with urgent/emergent indication for PCI, or with shock and hemodynamic instability were excluded as they usually have more severe disease presentation and complications, thus incurring higher cost. This definition is compatible with the definition of elective PCI adopted by other studies in the literature [11, 12]. This also enabled us to eliminate any likely operator-dependent variations as elective PCI is a fundamental, entry-level procedure for all cardiologists.

Clinical information included demographics, clinical presentation, angiographic severity, treatment details and in-hospital outcomes of PCI patients at the participating cardiac centres are captured via The Malaysian National Cardiovascular Database PCI Registry (NCVD-PCI) [5]. This online web registry was established in 2007 to record clinical data of PCI for performance appraisal and quality improvement purposes. Using this registry, we extracted clinical data of patients admitted to the TH and GH who underwent elective PCI during the study period.

### Cost data collection

By making necessary modifications to costing guidelines published in the literature [13], we devised a stepwise process for the cost data collection based on limitations in our medical and financial record keeping system. Primary cost data collection was conducted at various hospital departments for the time horizon of January 1^st^ to June 30^th^ 2014. The two main units of analysis were identified to be cardiac ward (CW) and cardiac catheterization laboratory (CL). Based on low complications rate reported in NCVD-PCI registry, it is taken as no serious complications occurred after elective PCI that require transfer to Cardiac Care Unit or Intensive Care Unit. The costing pathway ends upon patient discharge from the hospital.

**Fig 1:**
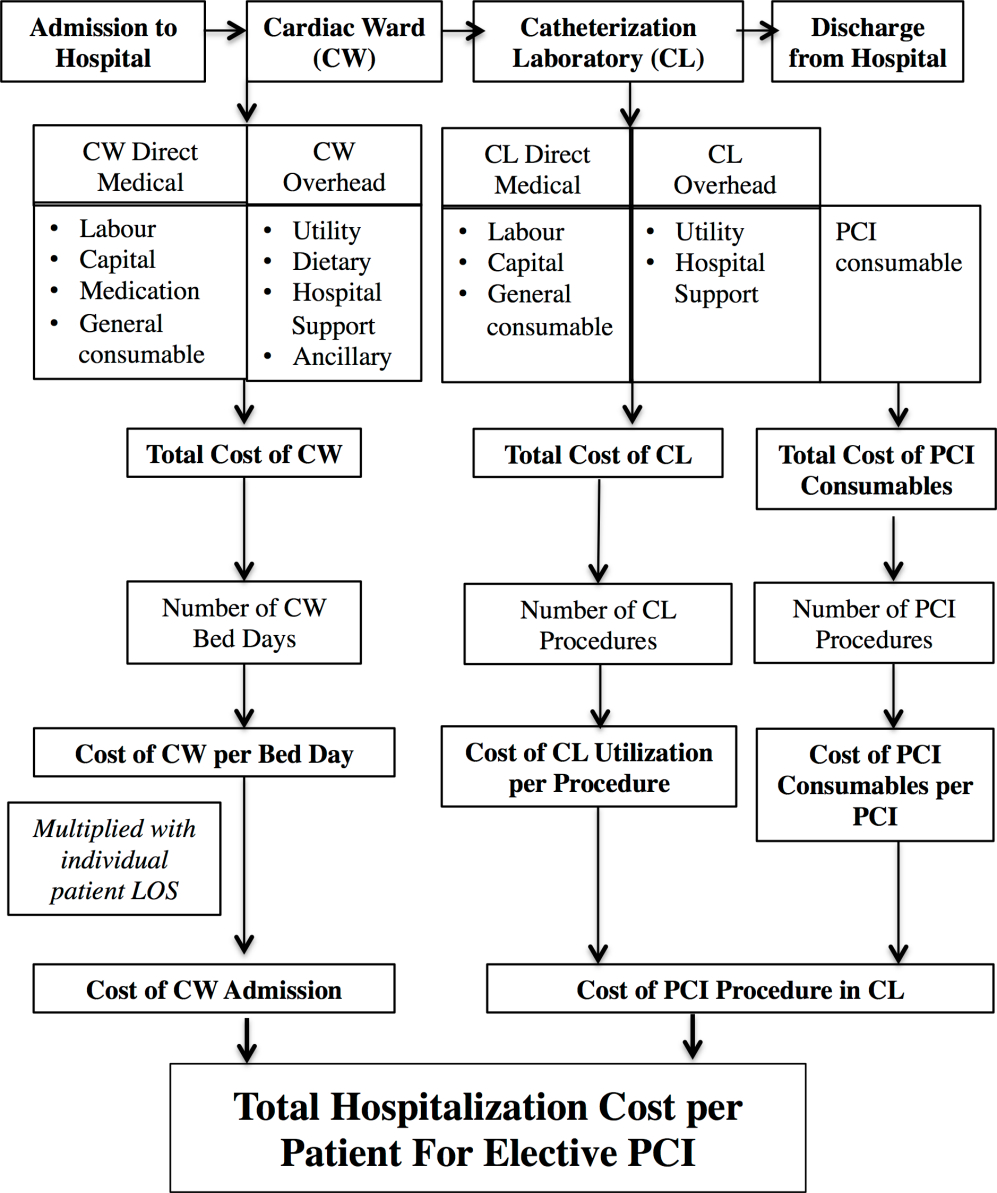
Pathway for the costing analysis of elective PCI.

Fig 1 illustrates the costing pathway of the mixed method applied in this study. Due to the time and resource constraint, a full bottom-up microcosting approach was not a feasible option. A mixed method combining top-down and bottom-up costing approaches was applied in order to obtain the unit cost of interest; namely CW admission cost and cost of PCI procedure in CL, which sum up as the total hospitalization cost for an elective PCI patient. Direct medical cost items included labour, capital, medication and general consumables. Overhead cost items included utility, dietary, hospital support and ancillary services. Using top-down approach, these costs were identified and valuated. A detailed description of the derivation and calculation of these cost items was outlined in a previous study [14]. A fully top-down costing approach only produced an average estimate of PCI hospitalization per patient. While this average cost enables an objective baseline comparison of resource consumption and hospitalization cost between the cardiac centres, it is insufficient for more comprehensive analysis such as patient-level comparison. Thus, bottom-up approach was used for cost item deemed to have significant impact on the final cost output, namely PCI-specific consumables. Actual quantity and purchase price of stents, guide wires, catheters and balloons used by each patient was obtained from the NCVD-PCI registry.

By dividing the total costs of CW and CL with appropriate activity output, unit costs of CW admission per bed day and per PCI procedure in CL were derived. By multiplying the cost of CW per bed day with the individual patient length of stay, the cost of CW admission per PCI patient can be added to cost per PCI procedure in CL to produce the total hospitalization cost per patient for elective PCI. All unit cost estimates were presented in the local currency, Malaysian Ringgit (RM), whereby USD 1 = RM 3.60 at the time of study.

### Statistical analysis

Cost data were tabulated with Microsoft Excel (2010) and merged with clinical data before being analyzed with IBM® SPSS® Statistics version 20.0.0. Continuous variables are presented as mean with standard deviation and compared with t-test. Categorical variables are presented as frequencies with percentages and compared with chi-square test. As for LOS and costs, non-parametric statistical test (Mann-Whitney) was used due to the skewed distribution of the data. Statistical significance is taken at p<0.05.

## Results

During the study period, 375 patients were admitted to TH and 240 to GH (Table 1). At both centres, patients were predominantly male. The mean age of patients was significantly higher in TH. The risk factors of CAD; namely dyslipidaemia, hypertension, obesity and smoking history were present in more than half of the patients in both centres. However, TH treated significantly more patients with diabetes mellitus compared to GH. As for cardiac history, there was significant difference between the 2 hospitals. TH received higher proportion of patients with documented CAD, recent onset of angina and previous PCI. Patients at TH also presented with worse symptom severity by NYHA and CCS classification. Furthermore, acute coronary syndrome was more prevalent among TH patients. On the contrary, more patients in GH suffered from previous myocardial infarction. They were likely treated with thrombolysis therapy, as numbers of previous PCI or CABG were low among GH patients.

**Table 1:** Comparison of patient characteristics at TH and GH.

	TH	n = 375	GH	n = 240	p value
	n	%	n	%	
**Demographics**					
Age, mean ± SD, years	60.1	10.1	58.2	10.6	0.029
Age>60	183	48.8	135	56.2	0.187
Male Gender	272	72.5	208	86.7	<0.001
**Risk Factors** ^**a**^					
History of Smoking	211	56.3	140	58.3	0.613
Dyslipidaemia	251	71.1	163	72.1	0.791
Hypertension	269	74.7	164	71.6	0.405
Diabetes Mellitus	183	54.0	73	32.0	<0.001
Body Mass Index ≥ 25	243	64.8	143	59.6	0.192
Family history of CVD	130	36.1	65	28.9	0.071
Cerebrovascular Disease	10	2.7	2	0.9	0.124
Peripheral Vascular Disease	1	0.3	1	0.4	0.726
Chronic Renal Failure	21	5.7	7	3.1	0.142
**Cardiac History**					
Documented CAD	185	52.1	78	35.0	<0.001
New onset angina	89	25.1	42	17.7	0.033
Previous Heart Failure	8	2.2	19	8.4	<0.001
Previous MI	119	33.2	91	42.9	0.021
Previous PCI	156	41.6	48	20.4	<0.001
Previous CABG	8	2.1	3	1.2	0.420
**Cardiac Status at Presentation**					
NYHA II-IV ^a^	92	25.0	35	14.7	0.002
CCS 2–4 ^a^	215	57.8	78	32.6	<0.001
Acute Coronary Syndrome	78	20.8	33	13.8	0.027

^**a**^ Risk factors, NYHA, CCS have missing data (<5%).

The percentages were determined from the available data.

Table 2 shows the comparison of PCI treatment details and outcomes. Radial approach was the preferred percutaneous entry. GH treated a significantly higher proportion of patients with multi-vessel disease, but TH treated a higher number of lesions per patient than GH. While the number of lesions with high-risk characteristics was generally low, TH recorded 4 times significantly more calcified lesion while GH had doubled the number of bifurcated lesions. Both centres had similar proportions of complex lesions type B2 and C based on the AHA/ACC lesion classification system. Overall, TH inserted more cardiac stents at a mean of 1.27 per patient compared to 1.16 at GH, even though the difference was not statistically significant. Drug-eluting stent was the most popular option at both centres whereas bioresorbable vascular scaffold was the least popular. Success rate was high and post-procedural complications were rare in both centres. Of the range of major adverse cerebrovascular or cardiovascular events (MACCE), only 2 cases of in-hospital mortality were recorded in GH. No incidence of other MACCE complications, namely post-operative stroke, post-operative acute myocardial infarction or non-elective re-intervention at both centres.

**Table 2:** Comparison of procedural details and outcomes at TH and GH.

	TH	n = 375	GH	n = 240	p value
	n	%	n	%	
**Medications**					
Thrombolytics	7	1.9	8	3.3	0.250
Glycoprotein 2b/3a	27	7.2	5	2.1	0.005
**Percutaneous Entry** ^**a**^					
Femoral	137	36.5	73	30.4	0.119
Radial	255	68	169	70.4	0.528
**Diseased Vessel** ^**a**^					
Left Anterior Descending	196	52.3	170	70.8	<0.001
Left Circumflex	107	28.5	62	25.8	0.464
Right Coronary	139	37.1	89	37.1	0.997
Left Main Stem	7	1.9	3	1.2	0.555
**Multi-vessel Disease**	56	14.9	66	27.5	<0.001
**Lesion treated per patient, mean±** **SD**	1.71± 0.81		1.53±0.76		0.007
**Lesion Characteristics**					
Ostial	8	2.1	10	4.2	0.144
Total Occlusion	13	3.5	12	5	0.348
Chronic Total Occlusion	26	6.9	8	3.3	0.057
Thrombus in lesion	9	2.4	4	2.2	0.797
Bifurcation	8	2.1	14	5.8	0.016
LMS	7	1.9	1	0.4	0.122
Calcified lesion	46	12.3	9	3.8	<0.001
**Complex Lesion by AHA/ACC**	216	57.6	132	55.0	0.263
**Stent Usage** ^**a**^					
Stents placed per patient, mean ± SD	1.27±0.79		1.16±0.66		0.072
DES placed per patient, mean ± SD	1.00±0.79		1.00±0.65		0.964
BMS placed per patient, mean ± SD	0.06±0.30		0.10±0.40		0.131
BVS placed per patient, mean ± SD	0.02±0.17		0.03±0.22		0.356
DEB placed per patient, mean ± SD	0.19±0.51		0.03±0.18		<0.001
**Treatment Outcomes**					
TIMI-3 post PCI	361	96.3	229	95.4	0.603
Successful Revascularization	354	94.4	231	96.2	0.299
In-hospital Mortality	0	0	2	0.8	0.077
Non-MACCE Complications	3	0.8	0	0	0.165

Note. DES = drug eluting stent, BMS = bare metal stent, BVS = bioresorbable vascular scaffold, DEB = drug-eluting balloon.

^a^ Each patient may have more than one percutaneous entry, diseased vessels and stents inserted.

In Table 3, the comparison between the length and cost of hospitalization were presented. While both centres recorded median LOS of 2 days, the mean LOS was longer in TH at 4.8 days compared to only 3.7 days in GH. This led to a significant difference in the ward admission cost. A quarter of the total cost can be attributed to CW admission (26.7% at TH and 25.4% at GH). Of the cost of PCI procedure in CL, PCI consumables cost was the major contributor. Overall, the cost of CW and CL were both significantly lower in GH (p<0.001 with Mann Whitney test). As a result, the mean total hospitalization cost in GH was lower at RM 12,117 (USD 3,366), compared to RM 16,289 (USD 4,525) at TH. Further scrutiny revealed that labour cost contributed to a higher percentage of CW and CL costs in GH as compared to TH. Another striking finding was that of the capital cost of CL in TH, which contributed to approximately two-thirds of the cost of CL utilization.

**Table 3:** Comparison of length of stay and costs of PCI at TH and GH.

	TH (n = 375)	GH (n = 240)		p value
**Length of stay (days)**				
Mean±SD	4.8±8.6	3.7±6.3		0.08
Median (IQR)	2.0 (2.0–3.0)	2.0 (1.0–3.0)		
**Cardiac ward admission cost**				
***Percentage of cost item***	***TH Ward***	***Ward***	***Daycare***	
*Labour*	*23*.*5%*	*42*.*1%*	*25*.*0%*	
*Capital*	*13*.*5%*	*6*.*1%*	*18*.*1%*	
*Consumable*	*4*.*1%*	*2*.*0%*	*3*.*4%*	
*Medication*	*37*.*2%*	*17*.*5%*	*4*.*5%*	
*Utility*	*3*.*1%*	*3*.*9%*	*4*.*5%*	
*Dietary*	*0*.*9%*	*1*.*8%*	*3*.*1%*	
*Hospital Support*	*2*.*9%*	*7*.*0%*	*8*.*0%*	
*Ancillary Service*	*14*.*8%*	*19*.*5%*	*33*.*4%*	
Mean±SD	4344.81±7809.66	3075.56±5123.15		<0.001
Median (IQR)	1813.36 (1813.36–2720.04)	1637.06 (818.53–2455.59)		
**Cost of PCI consumables**				
Mean±SD	8084.45±4329.29	7645.15±3551.71		<0.001
Median (IQR)	7299.91(5999.91–11099.91)	7535.57 (5135.57–7810.57)		
**Cost of PCI procedure in cardiac catheterization laboratory**^**a**^				
***Percentage of cost item***				
*Labour*	*21*.*2%*	*38*.*9%*		
*Capital*	*68*.*5%*	*34*.*4%*		
*Consumable*	*4*.*9%*	*19*.*9%*		
*Utility*	*2*.*8%*	*2*.*5%*		
*Hospital Support*	*2*.*6%*	*4*.*4%*		
Mean±SD	11906.44±4329.29	8993.27±3551.71		<0.001
Median (IQR)	11121.90 (9821.90–14921.90)	8883.69 (6483.69–9158.69)		
**Total hospitalization cost**				
Mean± SD	16289.17±8820.91	12117.45±6139.60		<0.001
Median (IQR)	13173.18 (11973.18–18173.18)	10569.37 (8854.59–14333.70)		

Note. All costs are in Ringgit Malaysia (RM). LOS and cost are not normally distributed. Levene's test of normality showed p<0.05. Mann Whitney test used to compare the mean differences.

^a^ Included cost of cardiac catheterization laboratory utilization and PCI consumables.

## Discussion

At present, two-thirds of the Malaysian population had at least one CVD risk factor whereas one-third had two or more risk factors. This phenomenon of cardiovascular risk factors clustering is shifting towards younger age group of Malaysian adults [15]. As a result, the economic burden of this cardiovascular epidemic is likely to escalate in the near future. Currently, the application of economic evaluation outcomes to health policy decision-making in Malaysia is limited by the paucity of cost evidence. Healthcare service expansion and financial planning require reliable procedure-specific clinical and cost information. Cost of medical procedures and hospitalization often depend on the type of hospitals. The cost differences may be due to the types of hospitals, the case mix of patients treated, management preference, and the outcomes of procedures. Using PCI, a common treatment modality for CAD, we explored the similarities and differences in terms of clinical presentation, treatment practice and hospitalization cost of cardiac service between a university teaching hospital and a general hospital. We found that patients who underwent elective PCI in TH had worse comorbidities and cardiac status on presentation compared to GH. In terms of procedural details, the number of lesions treated and the number of stents inserted were higher on average for patients in TH. They also stayed longer on average compared to patients in GH. Overall, the costs of cardiac ward admission, cardiac catheterization laboratory utilization and total hospitalization was higher for elective PCI patients in TH.

In many countries, university teaching hospitals provide undergraduate medical education and postgraduate specialty training for practicing doctors. These hospitals also serve as tertiary referral hospitals. Patients treated here are generally sicker on average, and the treatment approach tends to be more intensive and utilizing more advanced medical equipment, thus driving up the operation cost of the hospitals. For example, the cost of care per diem in a Swiss university hospital was found to be 3 times that of a non-teaching hospital [16]. Several studies, which examined the variations in hospital costs, concluded that among patients who underwent PCI, higher LOS and urban location of the hospitals predicted high hospital cost [17–19]. In a risk adjusted resource utilization study in Japan, the higher than expected cost of treatment for acute myocardial infarction was attributed to the teaching hospital status, with LOS being a strong predictor of hospital cost [20]. Similar findings can be reflected in our study. Apart from being an academic teaching hospital, TH is also located in the more urbanized capital city of the country and its PCI patients recorded a longer LOS when compared to GH.

Furthermore, underlying comorbidities on admission often have an impact on the eventual cost of CAD management. Many studies have reported that PCI patients who had diabetes mellitus often incurred a higher hospital cost, as a result of longer hospitalization period and increased resource utilization [21, 22]. Another study that compared PCI procedural cost between two hospitals showed that the hospital with more patients with adverse risk profile incurred a higher cost. The same hospital also recorded a higher number of stent inserted and lesions treated [23]. This was consistent with our study findings in which TH, the centre with a higher cost, received a significantly higher proportion of patients with diabetes mellitus and poor cardiac status.

Same-day discharge for selective PCI patients is a routine practice in GH. As a result, the ward admission cost was much lower in GH, partially attributing to a lower total hospitalization cost when compared to TH. Many previous studies have stated that same-day discharge for post-PCI patients may represent an important cost-saving strategy for both hospitals and society [24–26]. Reduction of LOS leads to less consumption of hospital resource per patient, the release of resources to benefit other patients, and eventual cost savings as a whole. Studies from different countries reached the same conclusion that selected low-risk PCI patients may be considered for same-day discharge, as there was no significant increased risk for death or readmission [27]. This recommendation was strengthened by findings from meta-analyses [28, 29]. By careful patient selection and establishing the necessary safety guidelines, same-day discharge for PCI patients can potentially bring down hospital costs without compromising patient safety.

From our findings, PCI consumables represented the largest component of total hospitalization cost at both centres. This is consistent with the findings of several international studies on hospital costing for PCI [30, 31]. However, in the course of this study, we found that the acquisition price of the stents differed between centres, even for the same brand of stents by the same manufacturing company. Such non-standardized medical device procurement practice is common in LMIC [32]. At present, there was no central purchasing agency of PCI consumables in Malaysia. In the face of rising healthcare costs, device acquisition needs to be guided by principles of quality care delivery and value for money based on clinical and cost effectiveness evidence, as well as value-based criteria such as equity. A viable option to control the consumable costs is to consider collaborative purchasing arrangement via centralized procurement scheme. Bulk purchasing before distribution to individual centres can lead to large discounts and this may eventually deliver cost reductions. A report released in England showed that discounts of 10–20% are possible of cardiac devices if the regional health foundation trusts joined together and engage with the market more effectively [33]. While this study did not provide a comparative analysis between different procurement practices at the study centres, we believe that current procurement process of PCI consumables should be reviewed to ensure greater value-for-money without compromising the flexibility and responsiveness in ordering and delivery. This is crucial to ensure a smooth delivery of service to patients all year long.

An increase in volume of PCI conducted in the cardiac centres was found to be associated with a decrease in adverse outcomes, length of hospital stay, and cost of hospitalization. In a nationwide study in United States, centres that performed >100 PCI procedures annually had significantly better outcomes, shorter LOS, and lower cost of hospitalization when compared with operators of low volume of annual procedures [34]. A meta-analysis on the same subject showed studies with larger sample sizes more often showed a relationship between operator volume and outcomes in PCI. Mortality and major adverse cardiac events increase as operator volumes decrease in PCI. However, the definition of high-volume operators varied with annual PCIs ranging from more than 11 to more than 270 cases, with no clear evidence of a threshold effect within the ranges studied [35]. In our study, there was no significant difference in the outcomes between the centres. The small sample size of only 2 centres and inclusion of only elective PCI patients might be the reason behind this, as poorer outcomes are more common among emergency PCI.

There are some limitations to our study. First, the cost findings cannot be generalized to other cardiac centres, especially those in the private sectors. Another limitation is the exclusion of cost incurred during pre-admission workup and follow-up period beyond the index hospitalization. The evaluation of long-term outcomes and associated costs is important to identify appropriate patients targeted for early or same-day discharge. Inclusion of these costs can be explored in future research to generate a more comprehensive overview of cardiac care provision. Despite its limitations, we believe that this study will serve as a starting point for further economic evaluation such as cost-effectiveness analysis or cost-utility analysis. To the best of our knowledge, this is the first comparative analysis of a cardiac procedure between teaching hospital and general hospital in Malaysia. Future research can be expanded to examine other types of cardiac services such as CABG and implantations of cardiac devices. This will guide the process of resource distribution and budget allocation in order to improve efficiency of cardiac service provision in Malaysia.

## Conclusions

With the advancement in medical technologies, healthcare costs will continue to escalate. The findings from this study provide an insight towards the variation in treatment practice and cost pattern for a common cardiac procedure at two public cardiac centres in Malaysia. Both types of hospitals are essential to provide equitable and high-quality health care system to the people and thus the identification of long-term sustainable financial strategies is vital. PCI patients at teaching hospital presented with higher risk, partially explain the higher cost of care. To offset this, cost saving strategies from general hospital could be incorporated into the clinical practice at TH to deliver better value for money. Same-day discharge at the day-care facility in general hospital led to shorter hospital stay and lower admission cost. In view of the high stent cost at both centres, cost effective consumable purchasing may result in substantial cost reduction. Comparison between centres provides vital information for decision-making in clinical practices, resource allocation and implementation of cost-saving strategies. Our study findings would serve as an impetus for further research in exploring the long-term outcomes and health economics of cardiac service in Malaysia.

## Supporting information

S1 File Patient dataset. (XLSX)
